# What is safe enough - asthma in pregnancy - a review of current literature and recommendations

**DOI:** 10.1186/s40733-018-0046-5

**Published:** 2018-12-27

**Authors:** Slavica Labor, Alba Maria Dalbello Tir, Davor Plavec, Iva Juric, Mihovil Roglic, Justinija Pavkov Vukelic, Marina Labor

**Affiliations:** 1 Faculty of Medicine, J.J. Strossmayer University of Osijek, Osijek, Croatia; 2 Department of Pulmonology, University Hospital Centre Osijek, Osijek, Croatia; 3 Institute of Public Health of Zagreb County, Zagreb, Croatia; 4 Children’s Hospital Srebrnjak, Zagreb, Croatia; 5 Department of Cardiology, University Hospital Centre Osijek, Osijek, Croatia; 6 Clinic for Pulmonary Diseases Jordanovac, University Hospital Cente Zagreb, Zagreb, Croatia; 7 Department of Internal Medicine, General Hospital Nasice, Nasice, Croatia

**Keywords:** Asthma, Pregnancy, Exacerbations, Adverse effect

## Abstract

**Background:**

Although asthma is one of the most serious diseases causing complications during pregnancy, half of the women discontinue therapy thus diminishing the control of the disease, mostly due to the inadequate education and fear of adverse events. Sadly, this is sometimes encouraged by insufficiently educated physicians. Since the incidence and the prevalence of asthma is increasing, it is important to arouse the importance of proper asthma therapy during pregnancy. Inadequate therapy, as well as interrupting or discontinuing therapy, may result in adverse perinatal outcomes for both mother and child.

**Main body:**

The main goal of asthma control during pregnancy is control of symptoms and prevention of exacerbations, same as in every asthmatic, but even more important. Maintaining optimal lung function, as well as regular daily activities, ensures maintenance of optimal fetal oxygenation. The therapy should be adapted depending on the frequency and severity of daily and nocturnal symptoms, demand for reliever therapy, by the limitations in everyday activities and the frequency of emergency asthma-related hospitalizations. Pre-conceptual education and therapy are very important and should be supported by an asthma action plan adjusted for the period of pregnancy. It is very important to note that most of the drugs used before pregnancy can be safely continued during pregnancy. Pharmacological and non-pharmacological therapy should be used in parallel. Pregnant women should be informed about the nature of the disease, therapy used during pregnancy, possible complications, avoidance of triggers, proper administration of therapy and, most important, why should the therapy be continued throughout the pregnancy on individual basis. Although drug treatment should be based on using drugs with less harm risk, if control of severe symptoms is needed to be achieved in order to protect both mother and child, any anti-asthmatic drug would have the beneficial benefit/harm ratio.

**Conclusion:**

There is no solid evidence that asthma treatment during pregnancy causes adverse outcomes for the mother and child but for many, especially new drugs, there is not enough data gathered. On the other hand, harmfulness of uncontrolled asthma during pregnancy is well documented so every effort should be put on preserving good control of asthma during pregnancy.

## Background

Asthma is a chronic respiratory disease caused by persistent inflammation and consequently bronchial hyperreactivity, airway obstruction and reduction of airflow. Exacerbations, usually caused by viral infections, and uncontrolled asthma result in hospitalizations and even fatalities [1, 2]. The incidence and prevalence of asthma is rising globally, bringing the total number to more than 300 million people with female predominance.

Among women, the disease is more common during gestational age, from 20 to 50 years. Additionally, complications, as well as hospitalizations are more frequent and more serious in women. According to some studies, this has been attributed to the level of female sex hormones, smaller diameter of airways and smaller lung capacity [2–4]. Changes in the disease severity have been seen with fluctuation of estrogen blood concentration during menstrual cycle [3] and incidence of exacerbations is higher in the year preceding pregnancy at the rate of 4.1% in future pregnant women [4]. Cortisol level elevation seen during pregnancy can protect from inflammatory triggers while increase in progesterone level may reduce the sensitivity of the respiratory tract to different provocative agents. Also, an increase in the levels of some bronchoconstrictors during pregnancy, such as prostaglandin F2α may cause bronchial obstruction [2].

Although asthma is one of the most serious diseases causing complications during pregnancy [1] a study showed that only half of the women continue their bronchodilator therapy in pregnancy while other half discontinue pharmacological treatment thus diminishing the control of the disease [4]. Mostly this is due to the inadequate education and fear that medical treatment can affect the health of the fetus but also due to perception that asthma will subside during pregnancy [1, 5]. It was found that asthma deterioration was observed in 36.3% of pregnant women, 33.6% women had an improvement while in 26.4% no change was noticed [5]. However, even in spite of education a drop in the use of drugs was confirmed, namely 23% reduction for the use of inhaled corticosteroids (ICS), 13% for short-acting β2-agonists (SABA) and 54% for oral corticosteroids (OCS) [6]. Many studies found that women with poorly controlled or uncontrolled asthma are more likely to give birth to a child of low birthweight, to have a premature birth or to have a birth via a caesarean section [7]. Also, some congenital malformations, like cleft lip and palate found in children, have been associated with both poorly controlled mother’s asthma or the use of systemic corticosteroids, primarily in the first trimester [7].

In general, morbidity and mortality in pregnant women with asthma is higher. Up to 45% of women experience asthma exacerbations during pregnancy, occurring most often between 24th and 36th week gestation [2, 7]. Studies focused on the impact of exacerbations and maternal hypoxia on the fetal development and occurrence of congenital malformations determined a significant association between severe asthma exacerbations in the first trimester of pregnancy and congenital malformations [7, 8].

Since the incidence and the prevalence of asthma, especially in women at childbearing age is increasing, it is of great significance to awaken the importance of proper asthma therapy during pregnancy [7]. Inadequate therapy as well as interrupting or discontinuing therapy may result in serious adverse perinatal outcomes for both mother and child. Taking this into account, observational and intervention studies about the safety of asthma therapy and interventions during pregnancy published during the last decade have been systematized and analyzed in this paper. Search for manuscripts in English was done through Cochrane Library, Medline, PubMed, ResearchGate and ClinicalTrials.gov using search terms: asthma, pregnancy, medication, education, and immunotherapy. Manual search through relevant journals, publications and guidelines was conducted with a search through reference lists of previously published articles on the topic. Only full texts of all articles found were considered relevant. Results of the search were combined and duplicates removed. The reviewed studies had to assess and measure an association between maternal exposure to antiasthma therapy (pharmacological, non-pharmacological or both) and possible adverse effects on pregnancy, perinatal outcomes and major birth defects. Methodological limitations of assessed studies were also considered. Some uncertainties identified during the systemic evaluation of the studies were discussed and resolved among the authors.

## Results from observational studies

For a long time, asthma and anti-asthma therapy during pregnancy have been associated with several adverse events affecting both the mother and the child, supported by different degree of evidence. Numerous congenital anomalies, including cleft lip or palate, heart malformations, spina bifida, congenital defects of the respiratory tract and anal atresia have been considered to be caused by anti-asthmatic therapy. Lin et al. analyzed data from the US multicenter case control study of the Birth Defects Prevention Study (NBDPS), which is still underway in 10 USA states, with thousands of respondents and referred to a section investigating the association of exposure to anti-asthmatic therapy with the occurrence of congenital anomalies [12]. Various outcomes were investigated: neural tube defects, esophageal, small intestine and anal atresia, defects of extremities, diaphragmatic hernia and omphalocele upon exposure to anti-inflammatory drugs, bronchodilators or their combination in the conception period. The conception period was defined as 1 month prior to pregnancy until the end of the first trimester of pregnancy. The study included 2853 children with one or more abnormalities and 6726 children without an anomaly as a control group. Mothers’ therapy was determined by an interview. After adjustment for confounders (sex of a child, age and BMI of the mother, number of deliveries, race, education level, alcohol consumption, smoking habit, folic acid and vitamin use, fever and crack/cocaine abuse), a significant association was found for isolated esophageal atrophy and exposure to bronchodilators (OR = 2.39, 95% CI 1.23–4.66), for anorectal atresia and anti-inflammatory therapy (OR = 2.12, 95% CI 1.09–4.12), and for omphalocele and combined use of anti-inflammatory drugs and bronchodilators (OR = 4.13, 95% CI 1.43–11.95). An increased risk for mothers using multiple bronchodilators was also detected, but this data was not published. However, after the analysis of the total number of malformations, both isolated and multiple in relation to these drugs, the only significant association was the one between omphalocele and combination therapy with anti-inflammatory drugs and bronchodilators (OR = 2.92, 95% CI 1.12–7.58). The authors pointed out that general prevalence for investigated malformations was as low as 1.2/10,000 births in 2003 for omphaloceles. Based on these data they concluded that the risk of asthma therapy causing omphalocele is only 0.05%. The limitation of this study is a lack of discrimination in determining whether malformations were the result of drug treatment, disease itself, recall bias or were just a coincidence. Both fetal hypoxia due to mother’s illness as well as recall bias may have the consequence of associating adverse pregnancy outcomes with asthma in pregnancy [12]. Study by Lim and Stewart included 33 differently designed studies conducted between 1974 and 2010 focusing on association between preventive anti-asthmatic therapy and outcomes for both the mother and the child [13]. One of the addressed studies in this review was the one by Blais et al. from 2009 showing the association between the use of ICS and congenital anomalies, mostly the ones of muscular and cardiovascular system. Data showed that women using > 1000 mcg/day of beclomethasone equivalent during the first trimester of pregnancy were 63% more likely to give birth to a child with congenital anomalies than women using < 1000 mcg/day or no treatment at all [14]. It is though unclear how ICS could cause various pregnancy adverse effects, as absorption of ICS to general circulation is low, and when absorbed their plasma concentrations is undetectable or low, or they are converted to a weak systemic CS. Studies in which they were used alongside OCS are exceptions [15, 16]. Only a study by Clifton et al. addressed the use of combination therapy in prevention of exacerbations and reported significant association between the use of fluticasone/salmeterol and the lower birth weight as well as length of the child compared to the use of budesonide [17]. The shortcoming of this study is a small number of included subjects with only 9 women in the group treated with combination therapy compared to 14 in the group on budesonide. This indicates that the disease itself rather than treatment has a negative effect on the pregnancy outcomes. Both the use of long-acting beta-2 agonists (LABA) and leukotriene receptor antagonists (LTRA) as preventive therapy during pregnancy, addressed in 7 and 5 systemized studies respectively is not sufficiently researched or documented. Plasma concentrations of LABA after inhalation, both salmeterol and formoterol are marginally detectable making their harmfulness very debatable. Several studies were conducted on the association of cromones with adverse events during pregnancy. In the study by Tata et al., a control group were children of mothers without asthma making it impossible to distinguish whether malformations were linked to prenatal exposure to cromones or to asthma itself [18]. Lim concluded, after systematization of 33 articles that no strong association between the use of maintenance therapy in asthma during pregnancy and congenital malformations can be established and that the decision to use maintenance therapy during pregnancy should be the result of carefully balancing between possible harm caused by uncontrolled asthma and potential adverse effects of therapy [13].

Some of the authors have restricted their findings to the first trimester of pregnancy. An international group of authors from Great Britain, Denmark and Norway has published a meta-analysis of three cohort studies conducted in Wales, Denmark and Norway on the association of mother’s use of anti-asthma therapy and congenital abnormalities [18, 19]. The results were a merger of the data from the European Register of Congenital Anomalies (EUROCAT) with the birth registers and registers of prescribed anti-asthma drugs in three countries stated above. There were 519,242 cases of birth, premature birth and termination of pregnancy in any gestational period due to fetal congenital anomalies analyzed. Out of those, 19,513 mothers were taking anti-asthma drugs and 650 children were born with various congenital abnormalities. Pre-exposure period was defined as 91 days before, 91 days after first pregnancy with at least two anti-asthma drug prescriptions taken during a year before pregnancy and birth. It was shown that a significant risk was present for most major congenital abnormalities after exposure to all types of therapy, OR = 1.21 (95% CI 1.09–1.34). Stratification to subgroups of congenital anomalies and types of drugs significantly increased the risk for anal atresia after exposure to ICS (OR = 3.4, 95% CI 1.15–10.04), similar to the results of the large USA study done by Lin. Also, an increased risk for major cardiac defects after exposure to combination of ICS/LABA (OR = 1.97), as well as for renal atresia after SABA exposure (OR = 2.37) was found. No significant association between the use of LABA and congenital anomalies has been detected. These results have been confirmed in other major studies [19]. In a Canadian study that included 13,280 pregnant women with diagnosed asthma and at least one prescription for an anti-asthmatic drug, an increased risk for most congenital abnormalities after exposure to the drug was confirmed (OR 1.34, 95% CI 1.22–1.47) [20]. A Swedish study by Kallen from 2014 that used Swedish Registry of Births also found that exposure to anti-asthma therapy during the first trimester of pregnancy increases the risk for most congenital anomalies, OR = 1.09 [21]. Blais, on the contrary, found the dose of ICS (> 1000 mcg of beclomethasone equivalent/day) that increased the risk for congenital abnormalities [14]. Since ICS therapy is the treatment of choice for asthma in pregnancy, along with LABA if needed, some authors conducted studies to determine the optimal dose of ICS. Cossette et al. [22] confirmed the relationship between low birth weight (less than 2500 g or below 10 percentile) or premature birth (prior to 37th week of pregnancy) and exposure to certain ICS doses or ICS/LABA combination. A cohort of 6199 women with asthma and their 7376 pregnancies was retrospectively analyzed from a healthcare database to a year prior to pregnancy and birth. The exposure to LABA in this group did not increase the prevalence of examined outcomes (OR = 0.81, 95% CI 0.58–1.12). An increase in prevalence was observed after isolated use of ICS at doses of > 125 mcg/day was examined, but this association was not significant. Higher doses of ICS may indicate also more severe forms of asthma, which can affect the outcomes. A significant association was confirmed for low birth weight (below 10 percentile), premature birth and low weight for gestational age with socioeconomic characteristics and age of women but authors failed to mention this in the discussion. Patients with low socioeconomic status had a significantly increased risk for all three adverse outcomes tested with OR = 1.8 for lower birth weight, 1.49 for premature birth and 1.45 for weight below 10 percentile. Pregnant women over 34 years had a significantly increased risk for premature birth with OR = 1.34, while women under 18 years had an increased risk for weight below 10 percentile (OR 1.58, 95% CI 1.01–2.46) [22]. Garne who concluded that age and socioeconomic status of the mother did not affect the results [19] contradicts these results.

Several studies focused on the impact of exacerbations and maternal hypoxia on the fetal development and occurrence of congenital malformations. The results were contradictory with most of the studies not detecting a significant effect and association of these variables. A study performed in the representative sample of 36,587 asthmatic pregnant women determined a significant association between severe asthma exacerbations in the first trimester of pregnancy and congenital malformations (severe exacerbations were considered those leading to hospitalization) [6].

The most extensive and comprehensive study on the effects of asthma on pregnancy itself and the outcomes of pregnancy was published in 2016 in Sweden [19]. The study was longitudinal, prospective, cohort, based on three Swedish national registers: Medical birth register, Prescribed drug register and National patient register. A cohort of 266,045 women and their 284,214 pregnancies was followed over a period of one year before pregnancy and until birth. Asthma, of varying intensity, both controlled and uncontrolled, was confirmed in 26,586 women (9.4%). It was established that asthma significantly increases (*p* < 0.001) the risk for almost all complications during pregnancy: preeclampsia, eclampsia, bleeding, premature contractions, premature membrane rupture and placental abruption. It also significantly increases (*p* < 0.001) the risk of low birth weight and small for gestational age. Upon stratification according to anti-asthma therapy, the results of the analyses did not changed significantly. The effects of the controlled and especially uncontrolled asthma on the outcomes of pregnancy were inconsistent. Authors concluded that asthma alone is the main factor complicating pregnancy and causing the adverse outcomes. This emphasizes the need to even more sensitize health professionals involved in pregnancy monitoring for more stringent surveillance and better control of asthma during pregnancy [23].

The analyzed observational studies have some limitations, depending on the design of the study itself. Samples tested for specific anomalies were small and the adherence to therapy was taken for granted although it was found that a large percentage of women, around 50%, quit the therapy during pregnancy [24]. Collecting data by interviews imposes recall bias and not all possible confounders (smoking, passive smoking, alcohol, environmental factors, socioeconomic status...) were registered. Large studies relying on registers are limited by the wrong classification of women, the inability to confirm compliance to the prescribed therapy, and the fact that there were unregistered confounding factors.

The conclusions made by most observational studies is that anti-asthma therapy increases the risk for some adverse events and complications of pregnancy with risk increasing with severity of the disease and dose of the treatment. It is not possible to ascertain whether outcomes are due to asthma itself or pharmacological therapy.

## Results from intervention trials

Given that the results of most observational studies have determined the increased risk for complications during pregnancy with a poor outcome, mostly for the child, interventional studies have been conducted and are still being carried out to unequivocally reveal risk factors. Addressed topics are types of anti-asthmatic drugs, safe dosage and route of administration of anti-asthmatic drugs that ensures the safest therapy and the best outcome for the mother and the child. Possibilities for a non-pharmacological therapy to be effective alone or with pharmacological therapy was also tested. Optimal therapy goals should decrease the incidence of exacerbations, attenuate the symptoms, avoid the complications of pregnancy, minimizing the risk of congenital abnormalities and improving the quality of life for pregnant women with asthma.

Acute asthma exacerbation is the most important event that can affect fetal morbidity and mortality during pregnancy. Thus, many studies have been conducted and are still underway seeking the optimal therapy that would reduce the severity of the disease and the incidence of exacerbations. Exposing pregnant women to any risk is unacceptable so studies could not be randomized in terms of pharmacological therapy. To circumvent this, authors have applied different approaches in their studies. Badawy did a small change in therapy proving that a drug is safe [25] while some authors have modified existing therapy after monitoring different parameters [26], or have made modifications in pregnant women after introducing non-pharmacological therapy [27, 28]. In one of the ongoing studies in South Australia, Grzeskowiak et al. are trying to determine whether enhanced antenatal care by a specially educated medical nurse in the Antenatal Asthma Management Service can lower the incidence of exacerbations in asthmatic pregnant women and if such antenatal centers are cost effective [27]. Results of this multicentric randomized controlled trial in 378 women are still not published, but the design of the trial underlines the importance of uncontrolled asthma in pregnancy. Other ongoing studies emphasize that pregnancy with asthma must have the best possible outcomes for the mother and the child. The Breathing for Life Trial, a multicentric randomized controlled trial, which is underway and relies on the Powell study described below, has multiple goals. One of them is to determine whether treatment adjustment guided by fraction of exhaled nitric oxide (FeNO) values can reduce adverse perinatal outcomes, decrease the incidence of exacerbations and reduce the incidence of bronchiolitis, croup and wheezing in infants. Other goals are the cost effectiveness of such protocol in everyday clinical practice and acceptance of such protocol by pregnant women, midwives and other care providers in antenatal clinical departments. The results have not yet been published [29]. Badawy conducted a randomized controlled trial of 60 respondents who had been treated at an emergency department intervening in the acute asthma therapy, with the aim of reducing the influence of exacerbations [25]. Half of the patients from control group received standard therapy (oxygen, iv. corticosteroid, iv. aminophylline and nebulized salbutamol), and the other half had the same therapy with a minor change. Magnesium sulphate was added in the nebuliser besides salbutamol. Patients have been followed up until delivery. Authors were prompted to do this intervention based on previous findings on the use of magnesium sulphate in gynecology and obstetrics and the treatment of severe adult asthma. Although the sample was small, the results were statistically significant showing reduction in the number of exacerbations, as well as significant improvement in lung function. Authors concluded that inhaled magnesium sulphate is a safe, accessible and inexpensive therapy for acute asthma in pregnancy [25].

In 2011, Powell published a study with a novel approach to treatment of asthma during pregnancy [26]. The hypothesis was that the intensity of symptoms does not always reflect the intensity and severity of inflammation in the airways, so the adjustment of anti-inflammatory and anti-asthma drug use based on the intensity of the disease does not provide optimal disease control and perinatal outcomes. In a randomized controlled trial, subjects were pregnant women, 12–20 weeks gestation, non-smokers, divided in two groups; controls (*n* = 103) and the intervention group (*n* = 100). The difference in the indications and adjustment of the required ICS and ICS doses together with LABA during the 2nd and 3rd trimesters of pregnancy was studied. The control group was monitored once a month, and adjustments of therapy was assessed and done after evaluation of symptoms and lung function using Asthma Control Questionnaire (ACQ). In the intervention group, a FeNO measurement was done adjacent to ACQ. The ICS dose was adjusted based on FeNO values, and when symptoms could not be adequately controlled, LABA was added. Results of the study showed significantly reduced incidence of exacerbations in the FeNO group (RR = 0.50, 95% CI 0.33–0.76, *p* = 0.001), improved quality of life (*p* = 0.037), less hospitalizations of newbors (*p* = 0.046), and lower exposure to ICS and OCS [26].

Several older studies of sufficient strength have been published on the use of non-pharmacological interventions for asthma control during pregnancy. In 2014, Zairina et al. identified initial 2387 studies and abstracts, but only three articles had sufficient strength for systematization and analysis [30]. Authors after systematization and analysis concluded that non-pharmacological therapy consisting of education, self-management, progressive muscle relaxation and periodic monitoring can help the control of asthma in pregnancy. More successful interventions included objective measurement of lung function or quantification of disease symptoms [30].

Nickel C et al. first applied progressive muscular relaxation (PMR) during pregnancy with asthma [31]. Muscle relaxation was already applied to patients with asthma with known benefits, but the effect of the use in pregnant women with asthma was not known. The aim of the intervention was to demonstrate that muscular relaxation improves many variables of both mother and child (blood pressure, lung function tests, heart rate variations, psychical status, quality of life, child birth weight). They recruited 64 subjects that were divided into two groups and were followed for 8 weeks with a control group receiving sham exercises. Authors showed that PMR improves FEV_1_ (*p* = 0.005) and PEF (*p* < 0.001), decreases heart rate fluctuations (*p* < 0.001) and significantly improves all examined mental health variables (anger, vulnerability, pain, social functioning, emotional health, mental health, overall health). The established benefits were explained by the increased activity of the parasympathetic nervous system triggered by PMR and participating in the control of breathing [31].

Murphy VE et al. examined if improving knowledge and skills through education and monitoring could improve the outcomes for mothers and infants [28]. Therefore, 211 asthmatic pregnant women were included into education performed by specially trained medical nurses in two sessions. First knowledge and skills test was conducted in 20th, and secondly in 33th week gestation. During the first visit to the antenatal center, it was established that 40% of pregnant asthmatics were not on continuous ICS treatment, 16% did not inhale properly, 42% did not have any knowledge of anti-asthmatics, only 15% had an action plan for asthma during pregnancy, and only 3% knew how to measure peak expiratory flow (PEF) properly. After the second visit, 21% of pregnant women were still not on continuous treatment, only 4% of the inhalation technique remained ungainly, the knowledge about drugs was present in 95% of pregnant women, the number of those with written action plan increased to 75, and 35% were able to measure PEF. Improvements in all areas were significant. However, no significant improvement in lung function, decrease in incidence of symptoms or the use of reliever therapy was observed. Women without an asthma action plan delivered female children with significantly lower birth weight compared to the group with the action plan (*p* = 0.043) with no difference found for newborn males (*p* > 0.05). Authors concluded that education improves outcomes and the course of pregnancy and should be part of the obstetric care in pregnancies with asthma [28].

Conclusions drawn by a small number of intervention trials are inconsistent not proving the efficacy of interventions for the successful outcomes of pregnancies or the quality of life of pregnant woman. In addition, intervention trials conducted so far were limited by small sample groups, poor design, short monitoring time and even lack of control group in some. Major trials are in process with large samples and carefully selected variables according to the priority outcomes of pregnancy for the mother and the child, results of which we should await.

## Discussion

This review presented recent studies of different design with the aim to determine the relationship between anti-asthmatic therapy and outcomes of pregnancy for both mother and child, indirectly determining the safety and efficacy of various anti-asthmatic drugs during pregnancy. Many observational studies and a small number of intervention trials have been evaluated. Studies of different designs have produced different conclusions. They differ in methodology (observation, intervention), sample size, interventions (drug, combination of drugs, non-pharmacological therapy), outcomes tested (congenital abnormalities, mental health of pregnant women, lung function of pregnant women, birth weight of child, incidence of exacerbations...), duration, inclusion of control group (some of them had inadequate or no control group at all), and the characteristics of the examined population (smokers, non-smokers). Results of most observational studies found a significant association between the use of ICS and some congenital anomalies [12, 14, 17, 19]. The association was stronger if mothers were exposed to higher doses of ICS or to a combination of ICS/LABA [12, 14, 17]. As many studies have found that asthma alone has an adverse effect on the outcome of pregnancy, a possible explanation for the found associations is that greater severity of the disease requires higher doses or combination of drugs. The conclusion that the severity of the disease is actually influencing the outcomes cannot be excluded [13, 14]. None of the studies found that LABA was associated with adverse effects if added to the ICS in case of ICS-resistant symptoms [13, 22]. Data associating LTRA or cromolyn exposure with adverse pregnancy outcomes is insufficient to make any conclusions [13]. None of the studies evaluated exposure to oral CS and outcomes of the pregnancy. Despite some positive association between different drugs and adverse pregnancy outcomes, authors of observational studies did not and could not, for the very design of the studies, determine whether this association was due to asthma, therapy, misclassification, recall bias or coincidence. The harmful effect of uncontrolled asthma on the other hand was determined in a largest cohort study [19]. Authors of the observational studies conclude that pharmacological control of asthma is required, and that ICS are the first choice of treatment because no strong link between ICS and adverse pregnancy outcomes has been demonstrated.

Asthma control should be individually approached and carefully accessed to distinguish the drug’s potential adverse effects against proven harmful effects of uncontrolled asthma [32]. This could also help in developing non-pharmacological methods that have been shown in several studies to have a positive effect on the overall quality of life of pregnant women. Additional treatment protocols like FeNO treatment adjustment added to standard care, and/or detailed education with improvement in knowledge and skills (like PMR), can improve outcomes [26, 28, 31, 33]. Non-pharmacological methods, in particular education, should become part of the standard antenatal care of pregnant women. Although four components of asthma education program were defined already back in 2003, they are still actual. They offer detailed information on asthma and treatment, regular self-monitoring of symptoms and/or peak expiratory flow rate measurement, regular physician’s checkup of disease severity and disease control. The fourth component is the possession of an individual asthma action plan in case of exacerbation of the disease [34].

## Current treatment recommendations

The main goal of asthma control during pregnancy is the control of symptoms and prevention of exacerbations, same as in every asthmatic, but even more important. Maintaining optimal lung function as well as regular daily activities ensures maintenance of optimal fetal oxygenation [1]. The therapy should be adapted depending on the frequency and severity of daily and nocturnal symptoms, demand for reliever therapy, limitations in everyday activities and frequency of emergency asthma-related hospitalizations. At the beginning of pregnancy women should be monitored once a week in order to assure stabile disease and therapy. Later controls should be once a month. Pre-conceptual education and therapy are very important and should be supported by an asthma action plan adjusted for the period of pregnancy. It is very important to note that most of the drugs used before pregnancy can be safely continued during pregnancy [1].

Pharmacological and non-pharmacological therapy should be used in parallel. Non-pharmacological therapy, not fully adopted yet in clinical practice implies patient education as well as asthma plan design. Pregnant women are informed about the nature of the disease, therapy used during pregnancy, possible complications, avoidance of triggers, proper administration of therapy and, most important, why should the therapy be continued throughout the pregnancy on individual basis.

Drug therapy for asthma during pregnancy resides on the same principles as for other asthmatic patients leveling up symptoms and risks against the treatment [2, 7]. Although drug treatment should be based on using drugs with less harm risk, when control of severe symptoms is needed to protect both the mother and the child, any anti-asthmatic drug would have the beneficial benefit/harm ratio. The level of treatment should be individually tailored using ICS as the basic treatment option with the stepwise (step-up or step-down) adjustments of dose and/or adding/stopping other maintenance treatment options [2, 35, 36]. Drug treatment should be complemented with regular assessments and non-pharmacological therapeutic options [2, 7].

Drugs available for pharmacological interventions in asthma include ICS and OCS, short and long-acting β2 agonists (SABA and LABA), leukotriene receptor antagonists (LTRA), theophylline, cromones and immunomodulators, like humanized monoclonal antibodies or allergen specific immunotherapy. Some of these drugs are used as maintenance treatment while others are rescue medication achieving immediate relief of symptoms (relievers). SABA are used in acute exacerbations providing fast bronchodilation and OCS can be used both as relievers and as maintenance therapy.

ICS are the most frequently and widely used therapy and several studies confirmed that ICS do not increase the perinatal risk of complications for either mother or child. They have the strongest safety profile for use during pregnancy with budesonide being the preferred ICS although the available data do not suggest that other ICS are more unsafe.

SABA are the preferred therapy in acute exacerbations with albuterol (salbutamol) as the treatment of first choice. LABA are used as an add on therapy when moderate doses of ICS do not lead to improvement of disease symptoms. Both SABA and LABA have similar pharmacology and toxicology profiles so it is expected that LABAs safety profiles resembles the one of albuterol. There are several LABAs prescribed in asthma but enough safety data has been gathered for these two: salmeterol and formoterol. Pregnancy safety data from human studies are lacking for newer LABAs, as indacaterol, olodaterol, and vilanterol. As in other asthmatics LABAs are only recommended as fixed combination with ICS.

Two leukotriene receptor antagonists (LTRA) are also available: zafirlukast and montelukast and they are in spite of limited available data both indicated for use during pregnancy [35].

Theophylline is used in mild persistent asthma as an alternative therapy. Due to many side effects and interactions with other drugs it is necessary to regularly control its serum concentration [9]. However, when used in asthma approach is different. The British Guidelines for Asthma Control from 2016 state that theophylline can be normally used in asthma, both through oral and intravenous application with a notion that it is necessary to reduce the therapeutic dose during pregnancy due to lower protein binding. Regular monitoring of theophylline blood concentration levels is necessary in women with acute severe asthma and when clinical presentation depends on the therapeutic doses of theophylline.

Use of OCS in pregnant women and the side effects have been subject of several trials but with contradictory results. Several studies showed association between the use of OCS and cleft lip or palate, maternal hypertension or preeclampsia and premature birth with low fetal mass. Yet many studies did not show any association of OCS with congenital malformations. Prednisolone is a drug of choice for oral use in pregnancy because it is metabolized by placental enzymes and only 10% of dose reaches fetus. According to the British Thoracic Society its use is recommended also during acute asthma attacks [10].

Omalizumab, a humanized monoclonal antibody that binds to circulating immunolglobulin E (IgE) [9], is one of the drugs approved for use in moderate to severe allergic asthma. A single descriptive study on 156 pregnant women did not detect any detrimental effects of omalizumab [11]. However, guidelines for asthma treatment during pregnancy briefly state that there are no clinical data on the use of omalizumab for moderate to severe allergic asthma [10]. Newer biologicals, anti-interleukin 5 monoclonal antibodies, including benralizumab, mepolizumab and reslizumab are still lacking human pregnancy safety data.

It is not recommended to initiate both subcutaneous (SCIT) or sublingual allergen specific immunotherapy (SLIT) during pregnancy due to the potential harm to the fetus as a systemic allergic reactions can occur, although the risk with SLIT is significantly lower but not negligible [37, 38]. On the other hand, patients tolerating maintenance SIT treatment and having benefits may continue its use.

Generally, it is recommended to graduate therapy according to the clinical presentation and skip the individual stages if there is clinical indication [2].

Acute asthma exacerbations are common during pregnancy. In about 20% of women, there is a need for medical intervention because of acute exacerbation and in approximately 6%, they are being admitted to hospital [39]. They and increase the risk of pre-eclampsia, gestational diabetes, placental abruption and placenta previa [40, 41]. The recommended pharmacotherapy of acute asthma exacerbations during pregnancy is not substantially different from the management in non-pregnant patients [42]. Intensive monitoring of both mother and fetus during such acute episodes is essential.

## Conclusions

There is no solid evidence that asthma treatment during pregnancy causes adverse outcomes for the mother and child but for many, especially new drugs, there is not enough data gathered, especially ones coming from randomized controlled trials. More information is available for the ICS and the combination of ICS and LABA, and less for other anti-asthmatics. On the other hand, harmfulness of uncontrolled asthma in pregnancy is well documented so every effort should be put on preserving good control of asthma during pregnancy. There is a constant need to evaluate the potential harm of therapy in relation to the proven adverse effects of uncontrolled disease and exacerbations. An individualized approach to treatment and interventions is suggested. Asthma education and regular follow-ups should become part of standard perinatal care with the emphasis on self-management. Health care professionals taking care of pregnant women need to be sensitized about asthma and importance of anti-asthmatic therapy. Every patient should be evaluated individually carefully taking in consideration risk of uncontrolled asthma compared to possible harm by the chosen therapy for both mother and child.

## Abbreviations

ACQ: Asthma Control Questionnaire
EUROCAT: European Register of Congenital Anomalies
FeNO: Fraction of exhaled nitric oxide
FEV_1_: Forced expiratory volume in 1st second
ICS: Inhaled corticosteroids
LABA: Long-acting beta-2 agonists
LTRA: Leukotriene receptor antagonists
OCS: Oral corticosteroids
PEF: Peak expiratory flow
PMR: Progressive muscular relaxation
SABA: Short-acting β2-agonists
SCIT: Subcutaneous allergen specific immunotherapy
SLIT: Sublingual allergen specific immunotherapy
